# The Acoustic Change Complex Compared to Hearing Performance in Unilaterally and Bilaterally Deaf Cochlear Implant Users

**DOI:** 10.1097/AUD.0000000000001248

**Published:** 2022-10-18

**Authors:** Jan A. A. van Heteren, Bernard M. D. Vonck, Robert J. Stokroos, Huib Versnel, Marc J. W. Lammers

**Affiliations:** 1 Department of Otorhinolaryngology and Head & Neck Surgery, University Medical Center Utrecht, Utrecht University, Utrecht, the Netherlands; 2 UMC Utrecht Brain Center, Utrecht University, Utrecht, the Netherlands; 3 Department of Otorhinolaryngology and Head and Neck Surgery, Antwerp University Hospital, Edegem, Belgium; 4 Department of Translational Neuroscience, Faculty of Medicine and Health Science, University of Antwerp, Antwerp, Belgium.

**Keywords:** Acoustic change complex, Cochlear implant, Frequency discrimination, Single-sided deafness, Speech perception

## Abstract

Clinical measures evaluating hearing performance in cochlear implant (CI) users depend on attention and linguistic skills, which limits the evaluation of auditory perception in some patients. The acoustic change complex (ACC), a cortical auditory evoked potential to a sound change, might yield useful objective measures to assess hearing performance and could provide insight in cortical auditory processing. The aim of this study is to examine the ACC in response to frequency changes as an objective measure for hearing performance in CI users.

**Design:**

Thirteen bilaterally deaf and six single-sided deaf subjects were included, all having used a unilateral CI for at least 1 year. Speech perception was tested with a consonant-vowel-consonant test (+10 dB signal-to-noise ratio) and a digits-in-noise test. Frequency discrimination thresholds were measured at two reference frequencies, using a 3-interval, 2-alternative forced-choice, adaptive staircase procedure. The two reference frequencies were selected using each participant’s frequency allocation table and were centered in the frequency band of an electrode that included 500 or 2000 Hz, corresponding to the apical electrode or the middle electrode, respectively. The ACC was evoked with pure tones of the same two reference frequencies with varying frequency increases: within the frequency band of the middle or the apical electrode (+0.25 electrode step), and steps to the center frequency of the first (+1), second (+2), and third (+3) adjacent electrodes.

**Results:**

Reproducible ACCs were recorded in 17 out of 19 subjects. Most successful recordings were obtained with the largest frequency change (+3 electrode step). Larger frequency changes resulted in shorter N1 latencies and larger N1-P2 amplitudes. In both unilaterally and bilaterally deaf subjects, the N1 latency and N1-P2 amplitude of the CI ears correlated to speech perception as well as frequency discrimination, that is, short latencies and large amplitudes were indicative of better speech perception and better frequency discrimination. No significant differences in ACC latencies or amplitudes were found between the CI ears of the unilaterally and bilaterally deaf subjects, but the CI ears of the unilaterally deaf subjects showed substantially longer latencies and smaller amplitudes than their contralateral normal-hearing ears.

**Conclusions:**

The ACC latency and amplitude evoked by tone frequency changes correlate well to frequency discrimination and speech perception capabilities of CI users. For patients unable to reliably perform behavioral tasks, the ACC could be of added value in assessing hearing performance.

## INTRODUCTION

Cochlear implantation has become a very successful treatment option for both adults and children with various degrees of sensorineural hearing loss, bilateral as well as unilateral. However, cochlear implant (CI) users show a large variability in speech perception performance (Blamey et al. 2013; Boons et al. 2012; Holden et al. 2013). The clinical measures to evaluate hearing performance depend on attention and linguistic skills (Mathew et al. 2017; Peelle 2018). Therefore, it is difficult to test CI users when they are unable to provide detailed or reliable feedback, especially poor-performing CI users and young children with a CI (Scheperle & Abbas 2015b; Sharma & Dorman 2006; Small & Werker 2012).

Psychophysical measurements such as frequency and spectral ripple discrimination might be an alternative since they do not require linguistic skills, and they have been found to correlate with speech perception in patients with sensorineural hearing loss (Papakonstantinou et al. 2011; Scheperle & Abbas 2015a) and CI users (Goldsworthy 2015; Kenway et al. 2015; Won et al. 2011; Zhang et al. 2019). Nevertheless, psychophysical tests still require attention and can be challenging to perform.

Therefore, objective measures are needed which reflect auditory performance, but do not require active participation or linguistic skills. One candidate is the acoustic change complex (ACC), which is an obligatory cortical auditory evoked potential (CAEP) evoked by a change in an ongoing stimulus (Jerger & Jerger 1970; Martin & Boothroyd 1999). It can be recorded in an awake and passive listening situation and it has the same typical waveform as the cortical P1-N1-P2 complex observed in response to a stimulus onset (Kim 2015; Martin & Boothroyd 2000; Vonck et al. 2019). The ACC mirrors auditory discrimination (Martin et al. 2008) and might better relate to perceptual measures (He et al. 2012, 2014; Kim 2015; Liang et al. 2018; Mathew et al. 2017) than other objective measures such as electrocochleography (Kim et al. 2017), electrically evoked compound action potentials (Smoorenburg et al. 2002; van Eijl et al. 2017), electrically evoked auditory brainstem responses (Lammers et al. 2015a), or CAEPs in response to onset stimuli (Barlow et al. 2016; Brown et al. 2015; Lammers et al. 2015b).

In normal-hearing (NH) and hearing-impaired listeners the ACC can be acoustically evoked by changes in pure tones, e.g. in frequency or intensity (Harris et al. 2007, 2008; Jerger & Jerger 1970; Pratt et al. 2009; Vonck et al. 2019, 2021), changes in natural speech tokens (Martin & Boothroyd 2000; Ostroff et al. 1998; Tremblay et al. 2003), or changes in more complex stimuli, like narrowband noise bursts with varying silent gap durations (Lister et al. 2007), or spectral-ripple stimuli (Won et al. 2011). More recently, several investigators examined the ACC in CI users and demonstrated that it can be reliably evoked by changes in frequencies within pure tones (Liang et al. 2018), speech stimuli (Brown et al. 2015; Friesen & Tremblay 2006), white noise stimuli with amplitude modulation changes (Han & Dimitrijevic 2020), or spectral-ripple stimuli (Won et al. 2011). The ACC can also be directly electrically evoked by changing the stimulation site from one electrode to another (i.e., spatial ACC; Brown et al. 2008; He et al. 2014; Hoppe et al. 2010; Kim et al. 2009; Mathew et al. 2017; Scheperle & Abbas 2015b). Interestingly, the ACC has been reported to correlate well with psychophysical measures (He et al. 2014; Mathew et al. 2017) and speech perception (Han & Dimitrijevic 2020; Liang et al. 2018; McGuire et al. 2021; Scheperle & Abbas 2015b) in CI users. Since frequency changes are essential components in natural sounds including speech, the present study will focus on the ACC evoked by frequency changes, which we previously studied in NH and hearing-impaired subjects (Vonck et al. 2019, 2021); the latter paper confirming correlations with speech perception.

In recent literature, CIs are discussed as a treatment option for late onset single-sided deafness (SSD) in order to restore binaural hearing (Arndt et al. 2017; Hansen et al. 2013; Mertens et al. 2017; Peters et al. 2021; van Zon et al. 2015). This new population of CI users with SSD offers a unique opportunity to study the processing of frequency changes in their CI ear and their NH ear, and to compare their frequency discrimination abilities with conventional bilaterally deaf (BD) CI users.

The main goal of this study was to examine the ACC in response to a frequency change as an objective measure for speech perception and frequency discrimination in both conventional BD-CI and SSD-CI users. Based on the recent literature (Han & Dimitrijevic 2020; He et al. 2014; Liang et al. 2018; Mathew et al. 2017; McGuire et al. 2021; Scheperle & Abbas 2015b), we hypothesize that ACC measures correlate with the perceptual outcomes. Secondary goals were to assess the differences in ACCs between BD-CI and SSD-CI users and perform within-subject analysis to compare the CI ears with the NH ears of the SSD-CI users. Considering the sustained overall cortical responsiveness caused by input of the NH ear to the auditory cortex in both hemispheres, we hypothesize that ACCs of the SSD-CI ears are larger than the responses of the BD-CI ears (Tillein et al. 2016) and that the NH ear shows larger ACCs than the CI ear of the SSD subjects. In the within-subject comparison between the CI and NH ear, we test whether the hearing performance with CI is based on central auditory and cognitive abilities, which are similar for both ears, or rather on the peripheral conditions, which differ between the CI and NH ear.

## MATERIALS AND METHODS

### Participants

Inclusion criteria were BD or SSD adults (age ≥ 18 and < 65 years) using a CI (Cochlear Ltd., Macquarie, NSW, Australia) for at least 1 year. We included only subjects aged below 65 years to diminish the possible effect of age on the ACC considering the cortical changes with age (Recanzone 2018). Among others, Harris et al. (2008) reported significantly higher ACC thresholds in adults aged 65 to 80 years who had normal pure-tone audiometric thresholds for the tested ACC frequencies (0.5 and 3 kHz) than adults aged 18 to 30 years. Exclusion criteria were neurological or mental disorders, or the use of anticonvulsant medication or psychotherapeutic drugs. The SSD-CI users were recruited from a randomized controlled trial currently running in the University Medical Center Utrecht (Peters et al. 2021). Single-sided deafness was defined as (near-)normal hearing in one ear (pure-tone average [PTA] 0.5, 1, 2, and 4 kHz ≤ 30 dB HL) and severe to profound hearing loss in the contralateral ear (PTA ≥ 70 dB HL).

Testing was in accordance with the Declaration of Helsinki (version 2013, Fortaleza) and the Medical Research Involving Human Subjects Act (WMO). The study was approved by the Medical Research Ethics Committee of the University Medical Center Utrecht (protocol number 16-558) and informed consent was obtained from all subjects. Participants received reimbursement for travel expenses.

Thirteen unilaterally implanted bilaterally deaf subjects (age between 19 and 62 years; 4 males) and six single-sided deaf subjects (age between 48 and 63 years; 1 male) participated in this study (Table 1). Median PTA of the six NH ears was 14 dB HL (range 5 to 21 dB HL). Full insertion was achieved in all subjects with various implant types from Cochlear Ltd. Four BD subjects were prelingually deaf, defined as severe to profound binaural hearing loss with its onset before the age of 2 years (based on medical charts including diagnostic audiometry and self-reported patient information) and insufficient residual hearing during childhood for normal speech and language development (Lammers et al. 2015a, 2015b). Duration of deafness ranged from 0.7 to 44.2 years, with a median of 7.2 years in the BD group and 1.8 years in the SSD group, which was not significantly different (Mann-Whitney *U* = 19, *p* = 0.08). Duration of implant experience ranged from 1.4 to 22.6 years, with a median of 12.0 years in the BD group and 1.7 years in the SSD group, which was significantly different (Mann-Whitney *U* = 2.0, *p* = 0.001).

**TABLE 1.** Subject characteristics

Subject	Sex	Age at Test (yrs)	Etiology	Age at Onset Severe to Profound HL (yrs)	Duration of Severe to Profound HL (yrs)	Implant Experience (yrs)	Implant Side	Implant Type*	Strategy
BD-01	M	62.3	Cochlear otosclerosis	57.6	0.7	4.0	Right	CI 422	ACE
BD-02	M	52.9	Hereditary (HMSN type I)	44.0	4.0	5.0	Right	CI 422	SPEAK
BD-03	M	22.8	Congenital	0.0	10.6	12.0	Left	CI 24R (CA)	ACE
BD-04	F	37.8	Meningitis	15.8	9.9	12.1	Left	CI 24R (CA)	ACE
BD-05	F	27.4	Meningitis	1.7†	13.2	12.5	Left	CI 24R (CA)	ACE
BD-06	F	49.3	Rubella	0.0	44.2	5.1	Left	CI 24RE	ACE
BD-07	M	52.5	Cogan’s syndrome	26.4	3.4	22.6	Right	CI 22M	SPEAK
BD-08	F	61.2	Hereditary (DFNA9)	57.9	1.0	2.3	Left	CI 422	ACE
BD-09	F	18.9	Congenital	0.0	2.5	16.4	Right	CI 24M	ACE
BD-10	F	53.3	Unknown	44.1	7.2	1.8	Left	CI 422	ACE
BD-11	F	62.0	Unknown	53.4	5.0	3.5	Right	CI 422	ACE
BD-12	F	49.0	Congenital	0.0	30.0	19.0	Left	CI 24M	SPEAK
BD-13	F	52.9	Unknown	25.0	12.3	15.6	Right	CI24R (CS)	ACE
SSD-01	M	60.2	Unknown	56.2	2.3	1.7	Right	CI 422	ACE
SSD-02	F	58.0	Cochlear otosclerosis	47.4	8.3	2.3	Left	CI 422	ACE
SSD-03	F	56.7	Sudden deafness	53.2	1.3	2.2	Left	CI 422	ACE
SSD-04	F	57.8	Sudden deafness	55.5	0.9	1.4	Left	CI 512 (CA)	ACE
SSD-05	F	48.1	Sudden deafness	42.2	4.3	1.6	Left	CI 512 (CA)	ACE
SSD-06	F	62.8	Sudden deafness	60.3	1.0	1.5	Right	CI 512 (CA)	ACE

* Each implant consisted of a multi-channel intracochlear array, with 22 electrodes numbered from base (no. 1) to apex (no. 22).

† Meningitis at the age of 1 yr with a deaf left ear and residual hearing in the right ear for a few years, which allowed her to achieve a good speech-language development (=marked as postlingually deaf).

BD, bilaterally deaf subjects (all unilaterally implanted); DFNA9, autosomal dominant genetic disorder causing inner-ear impairment; HL, hearing loss; HMSN type I, hereditary motor and sensory neuropathy (demyelinating disorder); SNHL, sensorineural hearing loss; SSD, single-sided deaf subjects.

### Study Procedures

All study procedures were conducted in a sound-attenuated booth. Subjects participated in one data collection session, requiring a total of 4.5 to 6.0 hours of participation. Frequent breaks were provided.

We assessed intelligence and musical experience in all subjects since these cognitive characteristics have been reported to have a positive effect on speech perception, frequency discrimination thresholds, and/or ACC measures (Parbery-Clark et al. 2009; Brown et al. 2017). To assess intelligence, all subjects underwent two subtests of the Wechsler Adult Intelligence Scale IV (WAIS-IV) (Silverstein 1985). The WAIS-IV is a validated full scale IQ test composed of 15 subsets covering four cognitive domains. In order to obtain an indicative IQ score of our study population, we used the vocabulary subset (verbal comprehension domain) and block design subset (perceptual reasoning domain). To assess musical experience, participants were asked if they practiced music and if so, how many hours a week and for how many years. We calculated a ‘musical experience score’ by multiplying the average amount of musical experience in hours per week by the years of active engagement. We considered a score > 15 to reflect significant musical engagement (Vonck et al. 2021).

#### Speech Perception

The subject’s own processor with their everyday map was used for all speech perception tests, since this reflects best their hearing. Speech perception in quiet and noise was measured using the Dutch Society of Audiology standard consonant-vowel-consonant (CVC) test (Bosman & Smoorenburg 1995) presented from a Yamaha MSP5 studio speaker (Yamaha Music Europe GmbH, Rellingen, Germany) located in front of the subject at a distance of 1 meter from the head. The speech stimuli were generated by Decos Audiology software (Version 2010.3.2, Decos Systems BV, Noordwijk, The Netherlands). Speech perception in quiet was assessed with the speech level fixed at 65 dB sound pressure level (SPL). Speech perception in noise was measured with a signal-to-noise ratio (SNR) of 10 dB: speech level fixed at 65 dB SPL with a stationary speech-shaped noise set at 55 dB SPL. Speech perception was scored based on the percentage of phonemes correctly repeated by the participant.

Speech perception in noise was also tested using the Dutch digits-in-noise (DIN) test, which consists of a list of triplets (three consecutive digits). The DIN test has shown to be applicable for determining the speech reception threshold (SRT) in patients with hearing aids and CIs (Kaandorp et al. 2015). All three digits had to be verbally repeated in the correct order by the subject for the triplet to qualify as correct. The stationary speech-shaped noise was set at 60 dB SPL. If the response was correct, the SNR for the next triplet was decreased by lowering the triplet level with 2 dB, and vice versa when incorrect. The SRT was defined as the SNR where on average 50% of the digit-triplets were repeated correctly. The DIN test was repeated once when the standard deviation (SD) exceeded 2.0. In that case, the DIN SRT with the lowest SD was accepted.

In the bilaterally deaf subjects, the ear contralateral to the CI was plugged with a disposable earplug over which an earmuff was placed (average noise reduction 32 dB; Howard Leight Viking V3, Honeywell, San Diego, CA, USA). In SSD subjects, we investigated both the CI ear and the NH ear in a randomized order. When testing speech perception with the CI ear, listening with the NH ear was prevented by presenting a continuous masking noise (International Speech Test Signal; Holube et al. 2010) via subjects’ personal in-ear headphones, over which an earmuff was placed. Speech perception in quiet was not tested in the NH ear.

#### Frequency Discrimination

For frequency discrimination, pure-tone stimuli were presented via custom-made scripts in MATLAB software (Version 7.11.0, MathWorks, Natick, MA, USA, 2011), a Creative® USB Sound Blaster HD sound card (Creative Technology Ltd., Jurong East, Singapore) and Decos Audiology Workstation (Patient Interface D2496-R, Decos Systems BV, Noordwijk, the Netherlands). The microphone of the CI processor was bypassed via the direct audio input cable of a research-dedicated speech processor (CP910, Cochlear Ltd.), identical to the study participants’ own processor with its everyday settings installed. For SSD participants, the NH ear was tested using a HD 200 audiometric headphone (Sennheiser Electronic GmbH, Wedemark, Germany).

We measured pure-tone frequency discrimination thresholds (FDTs) using a 3-interval, 2-alternative forced-choice, adaptive staircase procedure. Either the first or last tone differed from the other two and subjects were asked to indicate on a keyboard which tone was different. Feedback was not provided. Duration of each stimulus was 400 ms with cosine-squared onset and offset ramps of 5 ms, and a 300 ms silent interval. Stimuli were presented at the subjects’ comfortable sound level (commonly 75 dB SPL). The sound level was not changed between stimulus conditions within a subject. The test started with a large frequency difference (Δf), which was 68% of the reference frequency for CI ears and 2% for the NH ears. In case the subject discriminated the differences correctly, Δf was reduced (by a factor 2 and subsequently a factor 1.5), and vice versa, according to an adaptive up-down procedure. After 12 reversals the FDT was determined by averaging Δf for the last 6 reversals. The FDT was calculated as a percentage of the reference frequency.

The frequency discrimination procedure was performed three times. First, a short test round was performed at a frequency that differed from the tested frequencies (i.e., 1250 Hz) starting with a large Δf, to set the subject’s comfortable sound level and to check the subject’s comprehension of the test. Then, the procedure was performed twice in a randomized order, with a reference frequency that was centered in the frequency band of an electrode that included 500 or 2000 Hz, corresponding to the apical electrode (typically no. 20) or the middle electrode (typically no. 11). In order to compare performance between different stimulation sites and limit the burden of recordings to the subjects, we tested at two reference frequencies. For each subject, the individual’s frequency allocation table was used to determine these two reference frequencies. Supplemental Digital Content 1, http://links.lww.com/EANDH/B21 shows electrodograms according to the ACE speech coding strategy for stimuli as used in the frequency discrimination procedure and the frequency allocation tables of two subjects. Since pure tones activate multiple electrodes with different weights, small frequency changes (e.g., 2%) also are visible in the electrodogram.

#### ACC Stimuli

Individual sound stimuli were generated prior to the experiment using custom-made MATLAB scripts. For each subject, the same two reference frequencies as applied in the frequency discrimination test were selected (i.e., one centered in the frequency band of the middle, and one centered in the frequency band of the apical electrode). As also seen in the frequency discrimination test, stimuli were presented via an attenuator (Decos Audiology Workstation) and the direct audio input cable (bypassing the microphone) of the research-dedicated speech processor, identical to the study participants’ own processor with its everyday settings installed. The NH ear of the SSD subjects was tested using a TDH-39 headphone (Telephonics, Farmingdale, NY, USA) at a stimulus level of 75 dB SPL.

**Fig. 1.**
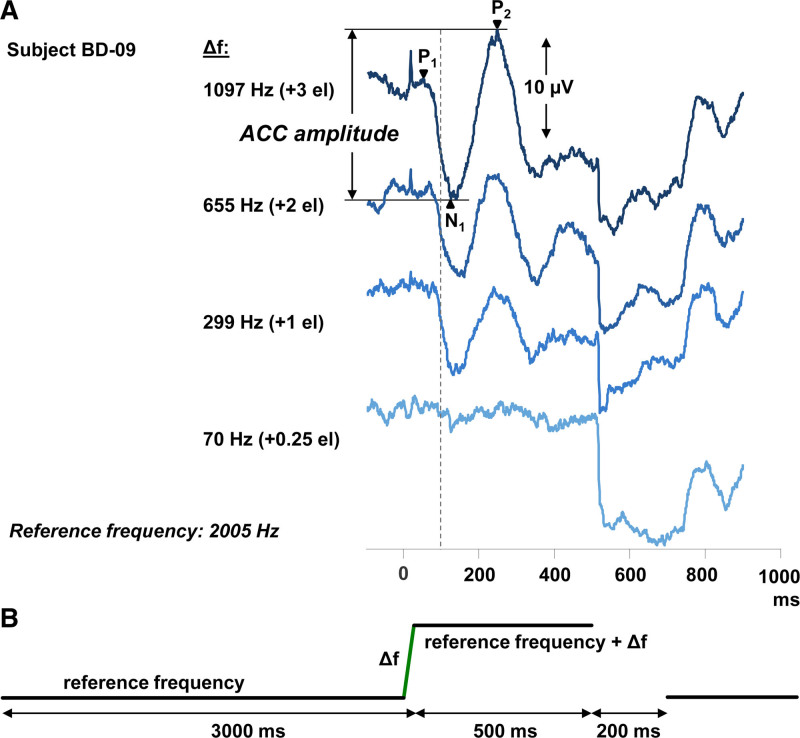
The acoustic change complex waveforms recorded from one subject (BD-09; A) along with the schematic presentation of the ACC stimulus (B). The stimulus consists of three components: (1) the reference frequency (~500 or 2000 Hz) with a duration of 2997 ms, (2) an upward logarithmic frequency modulation sweep of 3 ms with frequency change Δf, (3) a 500-ms target tone (reference frequency + Δf). The silent interval between stimuli was 200 ms. In this example (A), the reference frequency was 2005 Hz, centered in the frequency band of the middle electrode (no. 11). The vertical dashed line indicates 100 ms after the frequency change, the approximate latency where the N1 peak is expected. ACC indicates acoustic change complex; el, electrode.

The ACCs were evoked using pure tones of 3500 ms including a variable upward frequency change. The sound stimuli consisted of three components (Fig. 1), as described in Vonck et al. (2019): (1) the reference frequency with a duration of 2997 ms; (2) an upward logarithmic frequency modulation sweep of 3 ms with frequency change Δf; (3) a 500 ms target tone (reference frequency + Δf). We ensured that the second component started at the final phase of the first component and the third component started at the final phase of the second component, in order to prevent transient signals. The duration of the target tone was longer than in the previous study in order to avoid overlap of offset stimulus artefacts and the P2 peak. Cosine-squared onset and offset ramps of 5 ms were applied. The silent interval between stimuli was 200 ms. Time 0 of the recordings is defined by the onset of the frequency change.

For the CI ear, two reference frequencies with varying frequency changes of four different sizes were used, based on the individual frequency allocation table: a step within the frequency band of the middle or the apical electrode (further referred to as +0.25 electrode step), and steps to the center frequency of the first, second, and third adjacent electrode (further referred to as +1, +2, and +3 electrode step). The electrodograms of the stimuli are shown in Supplemental Digital Content 1, http://links.lww.com/EANDH/B21. This resulted in eight conditions per subject, which were presented in a random order. At the middle electrode, these steps corresponded to a frequency increase of about 5%, 15%, 35%, and 55% compared to the reference frequency, and at the apical electrode to an increase of about 5%, 25%, 50%, and 75%. We selected these four steps to explore which step would be optimal to obtain ACCs in each CI user, and to examine how ACCs vary with step size. Considering the wide range in age at onset of deafness and duration of deafness (Table 1) and assuming ACC thresholds will be higher for most CI users than in subjects with moderate hearing loss (1% to 10%; Vonck et al. 2021), the step sizes were assumed to be sufficiently large.

For the NH ear, the same individualized stimuli were used that were presented to the CI ear, but limited to the stimuli with frequency changes corresponding to +0.25 and +3 electrode steps, in addition to a stimulus with a frequency increase of 12% from the two reference frequencies, thus totaling six stimulus conditions. The 12% stimulus is also used in studies we are currently performing in NH subjects, which enables future comparisons between this group and the NH ear of SSD CI users. For consistency in reporting on the stimuli for the NH ear, we used the same description as for the CI ear. Thus, the stimuli with the reference frequency around 2000 Hz are referred to as the ‘middle electrode’ condition, and the stimuli with the reference frequency around 500 Hz are referred to as the ‘apical electrode’ condition.

#### ACC Recordings

Recordings were performed inside a Faraday shielded, sound-attenuated booth. Study participants were seated in a comfortable reclining chair and watched a muted documentary. They were carefully instructed prior to each recording to minimize movements and to fixate on the center of the video screen to minimize muscle and eye movement artefacts.

Electrophysiological responses were recorded by Ag/AgCl electrodes placed according to the 10 to 20 system using a Medelec Synergy T-10 Evoked Potential system. Recording electrode sites were at the vertex of the skull (Cz) and at the frontal midline (Fz). The mastoid contralateral to the stimulated ear was used as a reference electrode (A1 or A2). Eye movements and blinks were monitored using electrodes above and below the eye, contralateral of the stimulated ear. A ground electrode was placed off-center on the forehead.

Electrode impedance was kept below 4 kΩ for each electrode, with a between-electrode difference of less than 2 kΩ (regularly checked during the test session). The signals were recorded with a sampling rate of 50 kHz and filtered from 0.01 Hz to 100 Hz, while recording. Responses were acquired in a 1000 ms time-window, consisting of a pre-stimulus period of 100 ms (Fig. 1). In order to minimize contamination from eye-blinks or voltage excursions, responses containing amplitudes of more than 100 µV at any electrode were rejected and excluded from the averaged response. For each stimulus condition, 100 accepted recordings were averaged.

### Data Analysis

The Cz-A1 (or Cz-A2) recording was used to determine the presence or absence of an ACC, as well as to measure baseline-to-peak amplitudes and latencies. The first peak, P1, was considerably smaller than the following N1 and P2 peak. The low signal-to-noise ratio of this peak makes it difficult to reliably determine amplitude and latency of P1. Therefore, only the N1-P2 peak to peak amplitudes were analyzed. The N1 of the ACC was defined as the most negative peak at 70 to 170 ms after the onset of the frequency change. P2 was defined as the first pronounced positive peak occurring after N1 at 150 to 250 ms after the change (Fig. 1). For small frequency changes (+0.25 or +1 electrode step) latency shifts were allowed beyond these margins when the peaks agreed with peaks found at the larger frequency changes. The ACC amplitude was calculated as the difference between positive (P2) and negative (N1) peaks. We considered an ACC to be successfully evoked when the N1-P2 amplitude was greater than 4 µV or when on visual inspection an ACC waveform was unambiguous. Peak locations were manually identified in MATLAB by one investigator and checked by another. Disagreements were resolved by discussion. If no waveform could be identified, we marked it as no response for N1 and P2 latencies and assumed the ACC amplitude to be 0 µV (for nonparametric correlation analyses).

Statistical analyses were completed using SPSS software (version 25, IBM Corp, Armonk, NY, USA). We did not assume normal distributions of the data due to the small sample sizes. Friedman’s nonparametric test for repeated measures was used to analyze differences in averaged ACC latencies and amplitudes between the different stimuli. The nonparametric Kruskal-Wallis *H* and Mann-Whitney *U* test were used for comparing differences between independent groups. Wilcoxon’s signed rank test was used to analyze differences between paired groups (middle versus apical electrode, and NH ear versus CI ear of SSD subjects). The Spearman rank correlation was used to analyze the correlation between ACC variables (N1 and P2 latencies, N1-P2 amplitudes) and the speech perception scores and frequency discrimination. The stimulus condition with the most successful recordings was used in the correlation analysis. Correlation coefficients of *r* < 0.3 were considered as weak, *r* between 0.3 and 0.5 as moderate, and *r* > 0.5 as strong. Values with a *p*-value < 0.05 were considered statistically significant.

## RESULTS

### Hearing Performance

#### Speech Perception

Speech perception varied widely among all CI users (Table 2). The median score for correctly identified phonemes of CVC words in quiet was 85% (range 18% to 100%). The median score for correctly identified phonemes of CVC words in noise was 58% (range 0% to 82%) and the median digits-in-noise SRT was 0.4 dB (range −4.7 dB to 19.3 dB, n = 17 CI users). Two subjects (BD-07 and BD-13) did not perceive the digits in noise at all, so they were not able to complete the DIN test (for correlation analysis, SRT = 30 dB, the largest presented signal-to-noise ratio with still no correct response). The prelingually and postlingually deaf subjects did not significantly differ between each other in CVC-in-quiet scores, CVC-in-noise scores, and DIN SRT scores (Table 2; Mann-Whitney *U* > 19, *p* > 0.27). The BD-CI and SSD-CI groups did not significantly differ between each other in CVC-in-noise scores and DIN SRT scores (Mann-Whitney *U* = 28, *p* = 0.33, and U = 17, *p* = 0.11, respectively), so all CI ears were combined for further analysis with speech perception in noise.

**TABLE 2.** Speech perception, frequency discrimination, musical background and cognitive outcomes

	Subject	CVC-in-Quiet Score (%)	CVC-in-Noise Score (%)	DIN SRT (dB)	FDT Middle el. (%)	FDT Apical el. (%)	Musical Experience Score	Shortened WAIS Score
CI ear	BD-01	61	21	2.6	15.9	3.8	0	83
	BD-02	86	61	−0.3	4.3	2.7	0	120
	BD-03*	78	58	−3.4	2.2	5.2	4	70
	BD-04	97	70	−4.7	1.1	2.6	0	100
	BD-05	91	58	6.0	2.5	4.9	116	104
	BD-06*	30	21	8.7	13.9	20.0	0	49
	BD-07	45	27	30†	24.1	28.4	0	79
	BD-08	88	64	−1.0	1.3	2.4	13	81
	BD-09*	85	67	−4.1	3.4	6.3	0	70
	BD-10	91	67	0.1	9.5	6.0	7	69
	BD-11	91	76	−3.8	1.9	7.5	0	80
	BD-12*	58	30	11.4	6.3	9.4	0	52
	BD-13	18	0	30†	272†	272†	0	122
CI ear	SSD-01	45	39	6.5	3.1	3.5	30	68
	SSD-02	85	70	0.4	6.3	5.8	0	55
	SSD-03	100	82	−1.5	2.2	0.7	0	110
	SSD-04	85	70	1.0	8.3	2.8	0	121
	SSD-05	52	30	12.6	4.3	27.1	45	62
	SSD-06	48	30	19.3	3.9	47.9	0	69
NH ear	SSD-01	DNT	85	−5.5	0.3	0.7	30	68
	SSD-02	DNT	91	−7.0	1.2	1.2	0	55
	SSD-03	DNT	100	−7.6	0.7	1.5	0	110
	SSD-04	DNT	97	−6.4	0.7	1.1	0	121
	SSD-05	DNT	100	−8.5	1.6	1.5	45	62
	SSD-06	DNT	82	−6.0	2.1	2.7	0	69

* Represents prelingually deaf subjects.

† The largest presented signal-to-noise ratio or smallest presented frequency change as a % of the reference frequency with still no correct response. BD, bilaterally deaf subjects (all unilaterally implanted).

CVC indicates consonant-vowel-consonant phoneme score (% correct); DIN, digits-in-noise test; DNT, did not test; el., electrode; FDT, frequency discrimination threshold; NH, normal-hearing; SRT, speech reception threshold (dB); SSD, single-sided deaf subjects.

CVC-in-noise scores and DIN SRTs were strongly correlated (Tables 3 and 4; for all ears: Spearman’s *r* = −0.90, *p* < 0.001, and for all CI ears: *r* = −0.79, *p* < 0.001). Only three subjects had a musical experience score > 15, three subjects had minor experience (range 4 to 13), and the majority had no musical experience at all. The average scores of the two WAIS subtests reflecting intelligence varied from 49 to 122 with a median of 79. The BD and SSD groups did not differ with respect to these cognitive measures (Table 2). Speech perception scores were not related to the musical experience score (Spearman’s *r* < 0.09, *p* > 0.82) or the indicative IQ score (*r* < 0.38, *p* > 0.11).

**TABLE 3.** Speech perception and frequency discrimination correlations of all ears (NH and CI)

Spearman’s *r*	CVC in Noise	Digits in Noise	FDT Middle el.
CVC in noise	…		
Digits in noise	−0.90*	…	
FDT middle el.	−0.79*	0.82*	…
FDT apical el.	−0.79*	0.79*	0.76*

All NH and CI ears, n = 25.

* p < 0.001.

CI indicates cochlear implant; CVC, consonant-vowel-consonant; FDT, frequency discrimination threshold; NH, normal-hearing.

**TABLE 4.** Speech perception and frequency discrimination correlations of all CI ears (BD and SSD)

Spearman’s *r*	CVC in Quiet	CVC in Noise	Digits in Noise	FDT Middle el.
CVC in quiet	…			
CVC in noise	0.85*	…		
Digits in noise	−0.79*	−0.79*	…	
FDT middle el.	−0.63†	-0.61†	0.69†	…
FDT apical el.	−0.68†	-0.60†	0.66†	0.55†

All CI ears of the BD and SSD subjects, n = 19.

* p < 0.001.

† p < 0.01.

BD indicates bilaterally deaf; CI, cochlear implant; CVC, consonant-vowel-consonant phoneme score (% correct); el., electrode; FDT, frequency discrimination threshold; NH, normal-hearing; SSD, single-sided deaf.

For the NH ears of the SSD patients, median CVC-in-noise scores and DIN SRTs were 94% and −6.7 dB, respectively.

#### Frequency Discrimination

The CI ears showed a large variability in FDTs with a median of 4.1% (range 1.1% to 24.1%) for the middle electrode and 5.5% (range 0.7% to 47.9%) for the apical electrode (Table 2). One subject (BD-13) did hear the FD stimuli but was not able to discriminate the difference in frequency between the pure tones (for correlation analysis, FDT = 272%, the smallest frequency change with still no correct response). The prelingually and postlingually deaf subjects did not significantly differ between each other in FDT for the middle or apical electrode (Table 2; Mann-Whitney *U* > 20, *p* > 0.31). The BD-CI and SSD-CI groups did not significantly differ between each other in FDTs (Mann-Whitney *U* > 34, *p* > 0.89), so, as for speech perception in noise, all CI ears were combined for further analysis with the FDT.

**Fig. 2.**
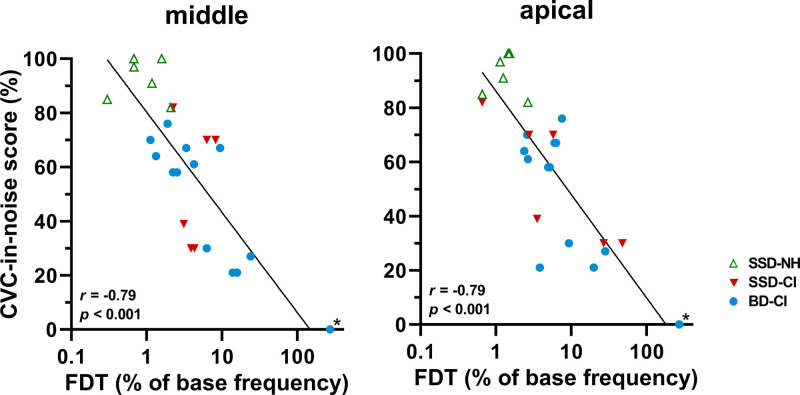
CVC-in-noise score (% of phonemes correctly repeated) as a function of FDT presented for the middle and the apical electrode condition. The line represents a linear regression and *r* is the Spearman rank correlation using combined data from all ears. *Represents subject BD-13 who did hear the FD stimuli but was not able to discriminate the difference in frequency between the pure tones (FDT = 272%, the smallest frequency change with still no correct response). BD-CI indicates CI ear of bilaterally deaf subject; CI, cochlear implant; CVC, consonant-vowel-consonant; FDT, frequency discrimination threshold; SSD-CI, CI ear of single-sided deaf subject; SSD-NH, normal-hearing ear of SSD subject.

Frequency discrimination was significantly correlated to speech perception in noise (Fig. 2, Tables 3 and 4). With a reference frequency at the middle electrode, FDTs were strongly correlated to CVC-in-noise scores (for all ears: Spearman’s *r* = −0.79, *p* < 0.001, and for all CI ears: *r* = −0.61, *p* = 0.005) and to DIN SRTs (for all ears: *r* = 0.82, *p* < 0.001, and for all CI ears: *r* = 0.69, *p =* 0.001). With a reference frequency at the apical electrode, FDTs were to a similar extent correlated to the CVC-in-noise scores (for all ears: Spearman’s *r* = −0.79, *p* < 0.001, and for all CI ears: *r* = -0.60, *p* = 0.006) and to the DIN SRTs (for all ears: *r* = 0.79, *p* < 0.001, and for all CI ears: *r* = 0.66, *p =* 0.002). So, in general, better frequency discrimination went along with better speech perception in noise.

Frequency discrimination thresholds were not related to the musical experience score (Spearman’s *r* < 0.30, *p* > 0.22) or the indicative IQ score (*r* < 0.43, *p* > 0.07).

The NH ears of the SSD patients showed a median FDT of 0.9% (range 0.3% to 2.1%) and 1.4% (range 0.7% to 2.7%) as percentage to the reference frequency of the middle and the apical electrode, respectively (Fig. 2). Note that the best-performing CI ears were in the range of the NH ears.

### Acoustic Change Complex

**Fig. 3.**
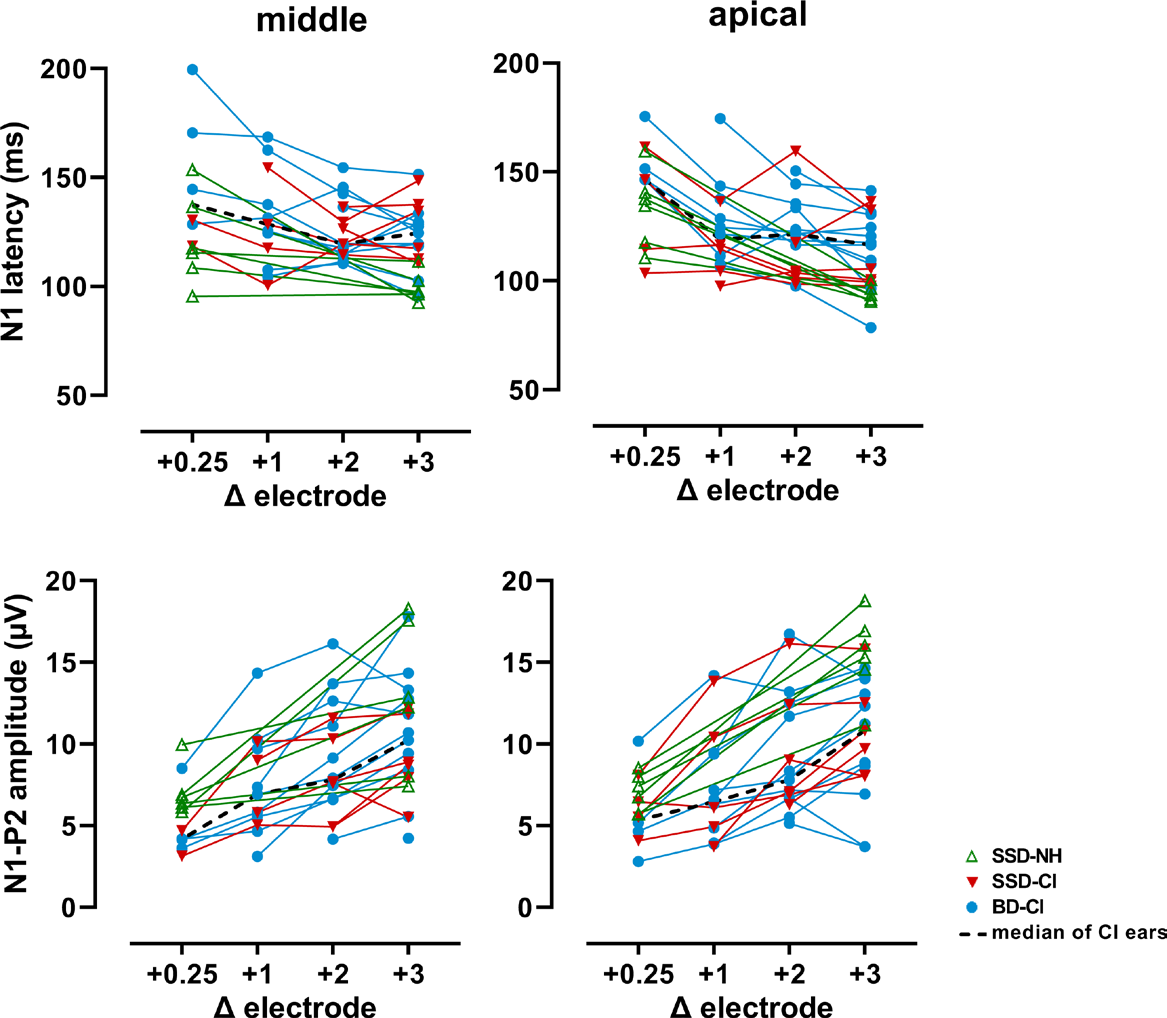
Individual N1 latencies and N1-P2 amplitudes as a function of the eight stimulus conditions: ∆ electrode +0.25, +1, +2, and +3 from the middle electrode (i.e., reference frequency around 2000 Hz) and apical electrode (i.e., reference frequency around 500 Hz). For the NH ear, the same stimuli were used that were presented to the CI ear, but limited to the stimuli with frequency changes corresponding to +0.25 and +3 electrode steps. Note that only data of the successfully evoked ACCs are shown. The dashed line indicates the median of the CI ears. ACC indicates acoustic change complex; BD-CI, CI ear of bilaterally deaf subject; CI, cochlear implant; SSD-CI, CI ear of single-sided deaf subject; SSD-NH, normal-hearing ear of SSD subject.

ACC responses exhibiting the typical N1-P2 waveform morphology were evoked in 17 out of 19 subjects (see for an example Fig. 1). Most successful recordings were obtained with the largest frequency change (+3 electrode step), with which we were able to record ACC responses in a maximum of 17 CI ears and in all six NH ears. Figure 3 shows the individual N1 latencies and N1-P2 amplitudes as a function of the eight stimulus conditions. In two subjects (BD-07 and BD-13), no ACC waveforms could be identified with this stimulus (N1-P2 amplitude was assumed to be 0 µV for nonparametric correlation analysis). Smaller frequency changes resulted in fewer successful recordings (+2 electrode step = 16 CI ears; +1 electrode step = 13 CI ears; +0.25 electrode step = 6 CI ears). Consequently, we used recordings evoked with the largest frequency change for correlation analysis.

For the CI ears, larger frequency changes resulted in shorter N1 latencies at the apical electrode (Fig. 3, Friedman’s *Q* = 18.5, *p* < 0.001), and larger N1-P2 amplitudes at the middle and apical electrode (*Q* = 14, *p* = 0.003, and *Q* = 21, *p* < 0.001, respectively). Focusing on differences between the ACCs at +2 and +3 electrode steps, the only statistically significant increase in amplitude was found with the middle electrode condition (Wilcoxon signed-rank test *Z* = −2.6, *p* = 0.011), whereas latency did not vary. For the NH ears, larger frequency changes resulted in shorter N1 latencies with the middle and apical electrode condition (Fig. 3, Wilcoxon signed-rank test *Z* = −2.0, *p* = 0.046, and *Z* = −2.2, *p* = 0.027, respectively), and larger N1-P2 amplitudes with the middle and apical electrode condition (*Z* = −2.2, *p* = 0.028, and *Z* = −2.2, *p* = 0.028, respectively).

We found no statistically significant difference between the CI ears of BD and SSD subjects in N1 latency (Mann-Whitney *U* > 1.0, *p* > 0.05) or in N1-P2 amplitude (Mann-Whitney *U* > 3.0, *p* > 0.48).

### ACC and Hearing Performance

**Fig. 4.**
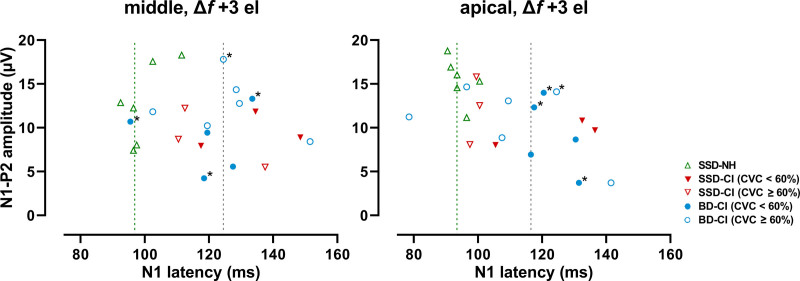
N1-P2 amplitude as a function of N1 latency in response to a stimulus with the largest frequency change (+3 electrode step). ‘Middle’ refers to the stimulus with the reference frequency around 2000 Hz. ‘Apical’ refers to the stimulus with the reference frequency around 500 Hz. Open symbols represent subjects with a CVC-in-noise score ≥ 60%, closed symbols represent subjects with a CVC-in-noise score < 60%. *Represents prelingually deaf subjects. The green dashed line indicates the median for the NH ears. The gray dashed line indicates the median for the CI ears. BD-CI indicates CI ear of bilaterally deaf subject; CI, cochlear implant; CVC, consonant-vowel-consonant; SSD-CI, CI ear of single-sided deaf subject; SSD-NH, normal-hearing ear of SSD subject.

Figure 4 plots N1-P2 amplitude versus N1 latency, roughly showing clusters of NH ears, CI ears of good performers (CVC-in-noise score ≥ 60%), and CI ears of poor performers (CVC-in-noise score < 60%). In general, the good performers (closed symbols) had latencies and amplitudes closer to those of NH ears (short latencies ~100 ms, large amplitudes ~10 to 15 µV) than poor performers (open symbols) who showed long latencies and/or small amplitudes.

**Fig. 5.**
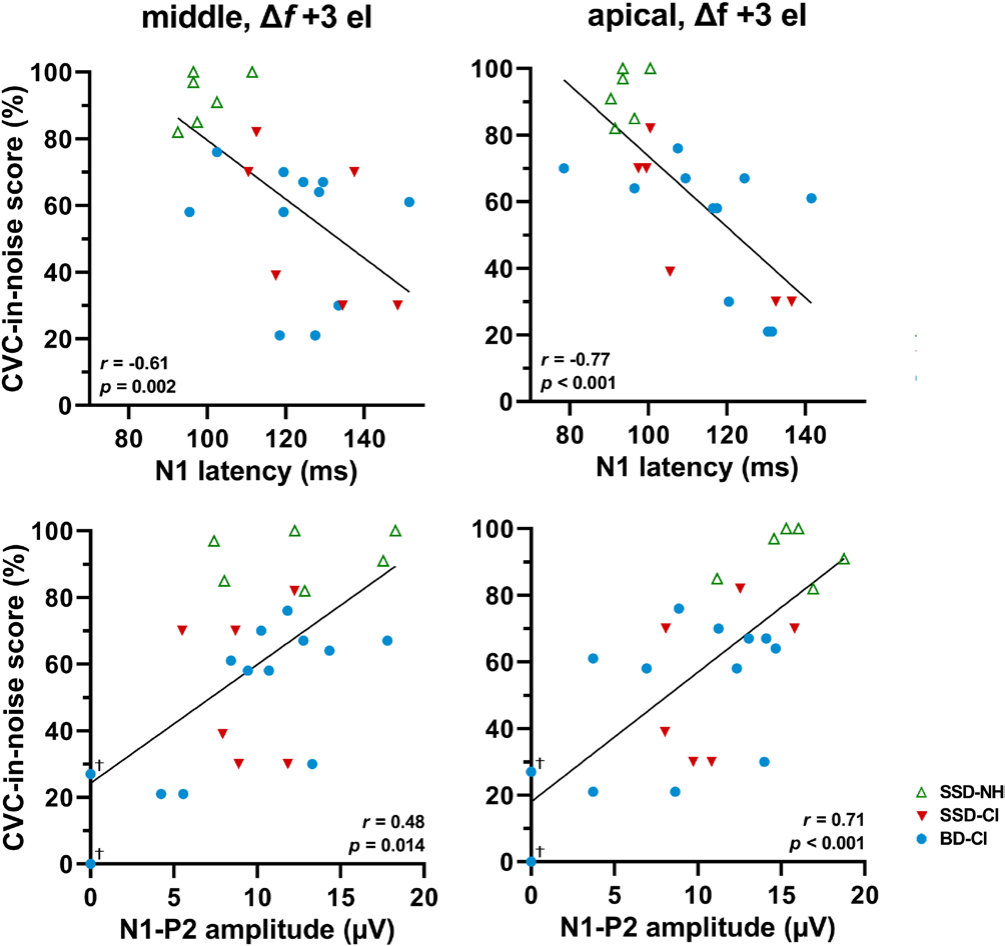
CVC-in-noise score (% of phonemes correctly repeated) as a function of the ACC latency and amplitude in response to a stimulus with the largest frequency change (+3 electrode step). ‘Middle’ refers to the stimulus with the reference frequency around 2000 Hz. ‘Apical’ refers to the stimulus with the reference frequency around 500 Hz. The line represents a linear regression and *r* is the Spearman rank correlation using combined data from all ears. †Represents subjects BD-07 and BD-13 with no successful ACC (amplitude of 0 µV). BD-CI indicates CI ear of bilaterally deaf subject; CI, cochlear implant; CVC, consonant-vowel-consonant; SSD-CI, CI ear of single-sided deaf subject; SSD-NH, normal-hearing ear of SSD subject.

Figure 5 shows the CVC-in-noise scores as a function of the ACC latency and amplitude for all ears including the NH ears of the SSD participants. With both the middle and apical electrode conditions, CVC-in-noise scores were negatively correlated to the N1 latency (Fig. 5, Spearman’s *r* = −0.61, *p* = 0.002, and *r* = −0.77, *p* < 0.001, respectively), and positively correlated to the N1-P2 amplitude (*r* = 0.48, *p* = 0.014, and *r* = 0.71, *p* <0.001, respectively). Digit-in-noise SRTs were correlated to ACC latencies and amplitudes to a similar extent (Table 5). When considering the correlations between CVC-in-noise scores and ACC measures for the CI ears only, correlations were less strong or not statistically significant (Table 6). Correlations with speech perception in noise (CVC in noise, DIN) and the P2 latency were weaker than with the N1 latency or not statistically significant (Tables 5 and 6).

**TABLE 5.** ACC correlations of all ears (NH and CI)

Spearman’s	N1 Latency				P2 Latency				N1-P2 Amplitude			
	Middle		Apical		Middle		Apical		Middle		Apical	
	*r*	*p*	*r*	*p*	*r*	*p*	*r*	*p*	*r*	*p*	*r*	*p*
CVC in noise	−0.61*	0.002*	−0.77*	<0.001*	−0.31	0.146	−0.51*	0.012*	0.48*	0.014*	0.71*	<0.001*
DIN	0.67*	<0.001*	0.70*	<0.001*	0.28	0.200	0.32	0.139	−0.53*	0.006*	−0.72*	<0.001*
FDT	0.61*	0.002*	0.72*	<0.001*	0.24	0.262	0.52*	0.011*	−0.47*	0.018*	−0.51*	0.010*

Correlations for ACCs evoked with the largest frequency increase (+3 electrode step). All NH and CI ears, n = 23 to 25.

* Significant correlations.

ACC indicates acoustic change complex; CI, cochlear implant; CVC, consonant-vowel-consonant phoneme score (% correct); DIN, digits in noise; FDT, frequency discrimination threshold; NH, normal-hearing.

**TABLE 6.** ACC correlations of all CI ears (BD and SSD)

Spearman’s	N1 Latency				P2 Latency				N1-P2 Amplitude			
	Middle		Apical		Middle		Apical		Middle		Apical	
	*r*	*p*	*r*	*p*	*r*	*p*	*r*	*p*	*r*	*p*	*r*	*p*
CVC in quiet	−0.18	0.496	−0.54*	0.025*	0.12	0.637	−0.39	0.127	0.56*	0.013*	0.46*	0.049*
CVC in noise	−0.31	0.225	−0.69*	0.002*	−0.01	0.964	−0.52*	0.033*	0.49*	0.034*	0.53*	0.020*
Digits in noise	0.40	0.113	0.48	0.051	−0.05	0.844	0.18	0.495	−0.53*	0.020*	−0.52*	0.024*
FDT	0.35	0.164	0.60*	0.011*	−0.18	0.479	0.39	0.124	−0.61*	0.006*	−0.27	0.271

Correlations for ACCs evoked with the largest frequency increase (+3 electrode step). All CI ears of the BD and SSD subjects, n = 17 to 19. ‘Middle’ refers to the stimulus with the reference frequency around 2000 Hz. ‘Apical’ refers to the stimulus with the reference frequency around 500 Hz.

* Significant correlations.

ACC indicates acoustic change complex; BD, bilaterally deaf; CI, cochlear implant; CVC, consonant-vowel-consonant phoneme score (% correct); DIN: digits in noise; FDT, frequency discrimination threshold; NH, normal-hearing; SSD, single-sided deaf.

**Fig. 6.**
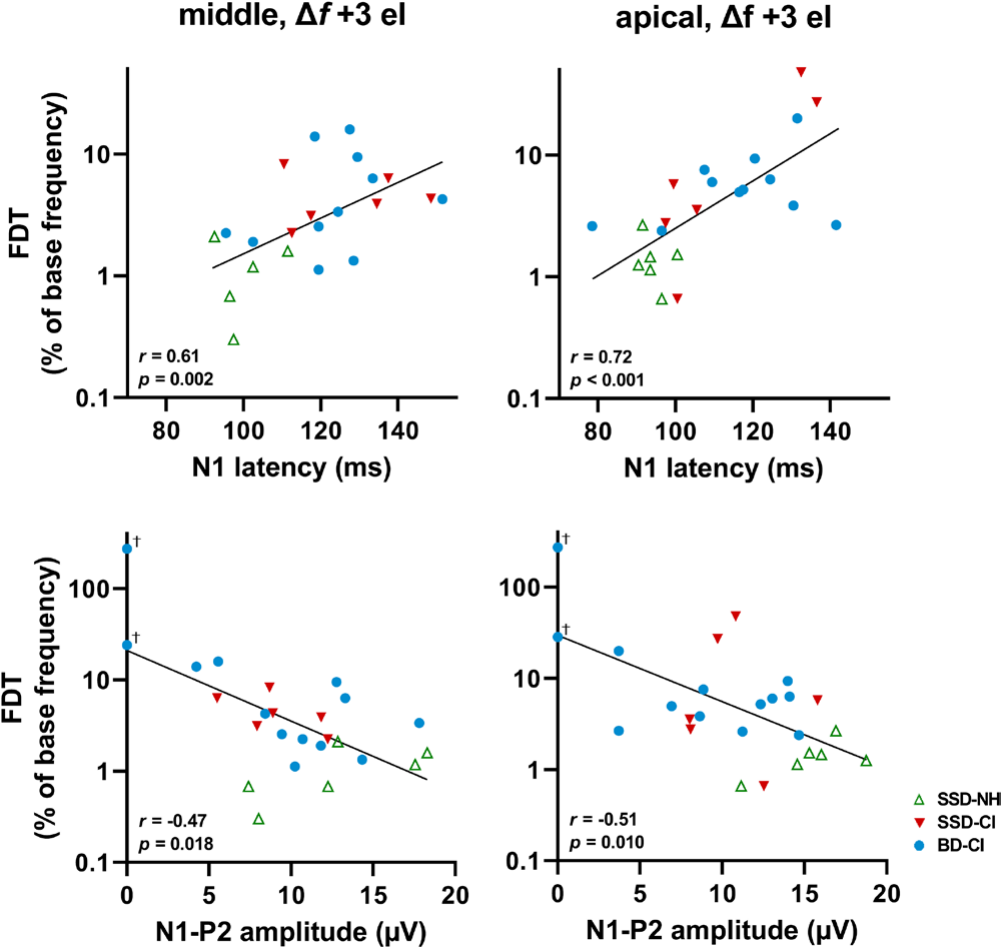
FDT as a function of the ACC latency and amplitude in response to a stimulus with the largest frequency change (+3 electrode step). ‘Middle’ refers to the stimulus with the reference frequency around 2000 Hz. ‘Apical’ refers to the stimulus with the reference frequency around 500 Hz. The line represents a linear regression and *r* is the Spearman rank correlation using combined data from all ears. †Represents subjects BD-07 and BD-13 with no successful ACC (amplitude of 0 µV). ACC indicates acoustic change complex; BD-CI, CI ear of bilaterally deaf subject; CI, cochlear implant; SSD-CI, CI ear of single-sided deaf subject; SSD-NH, normal-hearing ear of SSD subject.

With the middle electrode condition, the FDT was positively correlated to the N1 latency (Fig. 6, Spearman’s *r* = 0.61, *p* = 0.002) and negatively correlated to the N1-P2 amplitude (*r* = −0.47, *p* = 0.018). With the apical electrode condition, the FDT was positively correlated to the N1 latency (*r* = 0.72, *p* < 0.001), and negatively correlated to the N1-P2 amplitude (*r* = −0.51, *p* = 0.01). Correlations of the FDT with the P2 latency were considerably weaker than with the N1 latency (Table 5). When considering the correlations between FDT and ACC measures for the CI ears only, correlations were only significant for the N1 latency with the apical electrode condition (*r* = 0.60), and for the N1-P2 amplitude with the middle electrode condition (*r* = −0.61), as shown in Table 6.

In general, better speech perception and frequency discrimination corresponded to shorter N1 latencies and larger N1-P2 amplitudes.

### Comparison Between CI and NH Ears Within SSD Subjects

Speech perception We found no significant correlation between the DIN SRT (Table 2) of the NH ears and the CI ears (Spearman’s *r* = 0.31, *p* = 0.54), that is, the performance with the CI ear could not be well predicted from the performance with the NH ear.

#### Frequency Discrimination

**Fig. 7.**
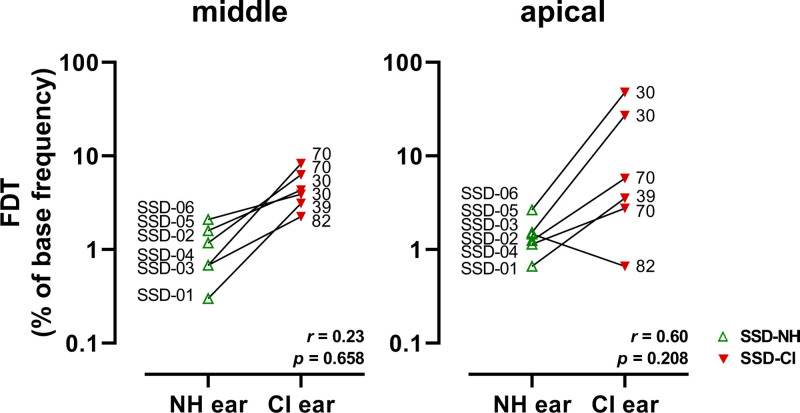
FDT of the NH ear and the CI ear of the SSD subjects. ‘Middle’ refers to the stimuli with the reference frequency around 2000 Hz. ‘Apical’ refers to the stimuli with the reference frequency around 500 Hz. Both ears of an individual subject are connected by a line. For each subject, the subject number is provided near the data point of the NH ear, and the CVC-in-noise score (% of phonemes correctly repeated) is provided near the data point of the CI ear. CI indicates cochlear implant; CVC, consonant-vowel-consonant; FDT, frequency discrimination threshold; SSD-CI, CI ear of single-sided deaf subject; SSD-NH, normal-hearing ear of SSD subject.

The median FDT of the six SSD-CI ears was 4.4 times higher at the middle electrode and 3.4 times higher at the apical electrode compared to the median FDT of the six SSD-NH ears (Fig. 7, Wilcoxon signed-rank test *Z* = −2.2, *p* = 0.028, and *Z* = −2.0, *p* = 0.046, respectively). Noticeably, FDTs widely varied at the apical electrode among the CI ears, with a threshold ranging from 0.7% to 48% of the reference frequency (3.3 to 240 Hz). We found no significant correlation between the FDT of the NH ears and the CI ears (Spearman’s *r* ≤ 0.60, *p* > 0.2), that is, the performance with the CI ear could not be well predicted from the performance with the NH ear.

Four out of six SSD subjects were able to discriminate frequencies within the frequency band of their middle or apical electrode. Remarkably, one subject (SSD-03) reached an impressive FDT of 0.7% with her CI ear, which was better than the FDTs of most NH ears, and slightly better than the FDT obtained with her own NH ear (1.5%, Fig. 7). With her CI ear, she also reached the best CVC-in-noise score (82%) of all CI ears and one of the best SRTs with the DIN test (−1.5 dB).

#### Acoustic Change Complex

**Fig. 8.**
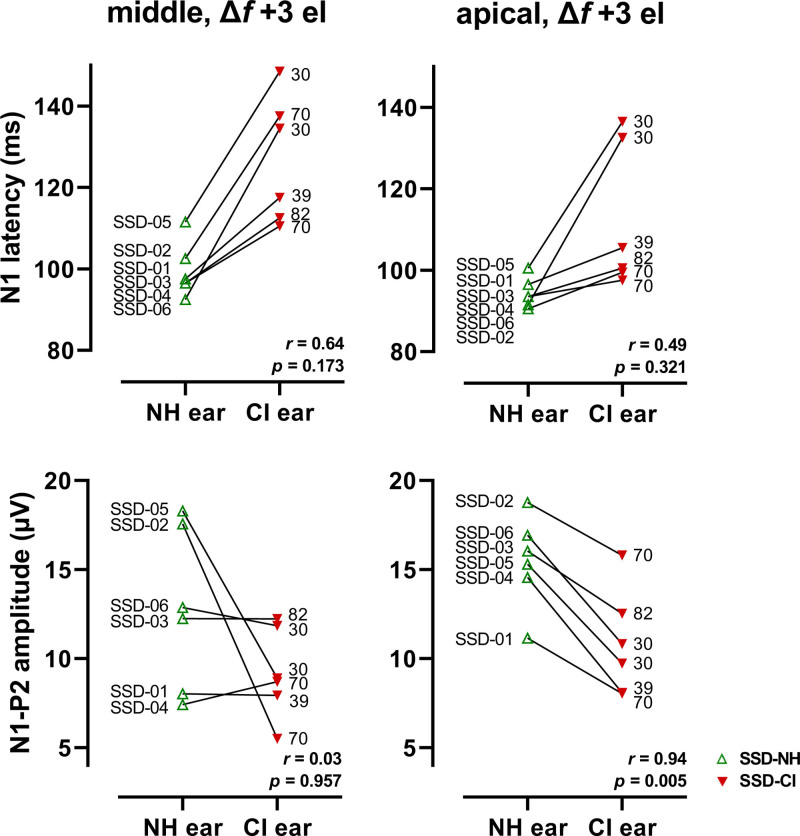
Individual N1 latencies and N1-P2 amplitudes of the NH ear and the CI ear of the single-sided deaf subjects in response to a stimulus with the largest frequency change (+3 electrode step). ‘Middle’ refers to the stimuli with the reference frequency around 2000 Hz. ‘Apical’ refers to the stimuli with the reference frequency around 500 Hz. Both ears of an individual subject are connected by a line. For each subject, the subject number is provided near the data point of the NH ear, and the CVC-in-noise score (% of phonemes correctly repeated) is provided near the data point of the CI ear. CI indicates cochlear implant; CVC, consonant-vowel-consonant; SSD-CI, CI ear of single-sided deaf subject; SSD-NH, normal-hearing ear of SSD subject.

When we compared the ACCs of the six CI ears to the six NH ears within the SSD subjects, we found shorter N1 latencies and larger N1-P2 amplitudes for the NH ears (Fig. 8). The median difference in latency was 29 ms for the middle electrode condition (i.e., reference frequency around 2000 Hz) and 9 ms for the apical electrode condition (i.e., reference frequency around 500 Hz; Wilcoxon signed-rank test, *Z* = −2.2, *p* = 0.028, and *Z* = −2.2, *p* = 0.027, respectively). The median difference in amplitude was 3.8 µV for the middle electrode condition and 5.4 µV for the apical electrode condition, only statistically significant for the latter (Wilcoxon signed-rank test, *Z* = −1.4, *p* = 0.17, and *Z* = −2.2, *p* = 0.028, respectively). We found a significant correlation between the ACC amplitude of the NH ears and the amplitude of the CI ears with the apical electrode condition (Spearman’s *r* = 0.94, *p* = 0.005), but not with the middle electrode condition (*r* = 0.03, *p* = 0.96). There was no significant correlation between the N1 latency of the NH ears and the latency of the CI ears for either reference frequency (*r* ≤ 0.64, *p* > 0.17).

Subjects SSD-02 and SSD-05 showed a large difference in amplitude between their CI ear and NH ear for the middle electrode condition (Fig. 8). On visual inspection, this was not associated with their speech perception in noise. Note that they both had the longest duration of deafness in the SSD group (Table 1, 8.3 and 4.3 years, respectively). Subjects SSD-05 and SSD-06 showed a large difference in latency between their CI ear and NH ear for the apical electrode condition. They both had the worst speech perception scores in noise in the SSD group (Table 2, CVC-in-noise scores 30% and 30%, and DIN SRT 12.6 dB and 19.3 dB, respectively).

## DISCUSSION

The main goal of this study was to examine the ACC as an objective measure for hearing performance in CI users, by correlating ACC measures with speech and frequency discrimination. Our results show that, both in traditional bilaterally deaf CI users and in single-sided deaf CI users, the N1 latency and N1-P2 amplitude were moderately to strongly correlated to hearing performance. CI users with good speech perception and frequency discrimination had shorter N1 latencies, with larger N1-P2 amplitudes. Thus, the ACC can be applied for CI users as an objective measure of hearing performance.

We hypothesized that ACCs evoked by CI stimulation in SSD subjects would be larger than in BD subjects, but we found no difference in ACC outcomes between either group. As expected, the CI ears of the SSD subjects showed longer latencies and smaller amplitudes than their contralateral NH ears.

### ACC and Speech Perception

The relationship between ACC latency and speech perception is supported by two recent studies in bilaterally deaf CI users. Liang et al. (2018) evoked ACCs in 12 CI users (18 CI ears), in response to frequency changes within 1-s tones. They reported that the N1 latency was strongly correlated to speech perception in quiet (Pearson’s *r* = 0.60, *p* < 0.05). In a more recent study (McGuire et al. 2021) the same research group showed similar results in 21 CI users (29 CI ears) using the same change stimuli (r = 0.40, *p* < 0.05). Han & Dimitrijevic (2020) evoked the ACC in ten CI users in response to amplitude modulation (AM) changes in white noise stimuli at AM rates of 4, 40, 100, and 300 Hz. In line with our results, the N1 latencies for the poor CI performers (speech perception score in noise < 50%) were delayed compared to the good CI performers. Strong correlations were found between the N1 latency for the 40 Hz AM rate and various speech perception measures, including vowel perception in quiet and in noise (Spearman’s *r* = −0.75, *p* < 0.05; *r* = −0.84, *p* < 0.05, respectively).

With respect to the ACC amplitude, findings reported in literature are less consistent. The results of our study indicating that subjects with poor speech perception have smaller amplitudes are supported by Scheperle & Abbas (2015b). They applied two different change stimuli, both with a 800-ms duration: one by changing the stimulation site from one to another electrode (always including electrode no. 12 of 22) and one by reversing the spectrum of a rippled noise. For both stimuli, they found a strong correlation with speech perception in noise (n = 11 CI recipients). In contrast to these findings, Brown et al. (2015) using 800-ms vowel-change stimuli found no predictive relationship between the ACC amplitude and speech perception in noise in a group of ten Nucleus^®^ Hybrid CI users with residual low-frequency acoustic hearing. McGuire et al. (2021) also reported that the N1-P2 amplitude did not correlate with speech perception outcomes. Similarly, Mathew et al. (2017) using 800-ms electrode-switch pulse-train stimuli did not find a correlation between the ACC amplitude and speech perception in quiet at 1 week post-activation in ten bilaterally deaf CI users, even after excluding the three prelingually deafened individuals from the analysis. They reported that the study was underpowered and that the poor correlation was likely due to the large inter-subject variability in ACC amplitudes (Mathew et al. 2017), which is consistent with our own and other observations indicating substantial variability in amplitudes (e.g., He et al. 2012; Liang et al. 2018).

The difference in outcomes of the studies may partially be explained by the difference in CI experience of the subjects and stimuli that were used, especially with regards to the duration of the reference tone. In Mathew et al. (2017), CIs were recently activated, whereas CI experience was more than one year in the other studies (Scheperle & Abbas 2015b; Brown et al. 2015; current report). Interestingly, in the follow-up study at 1-year post-activation, Mathew et al. (2018) reported that the development of the spatial ACC preceded accurate behavioural discrimination. Within the 800-ms stimulus used by Mathew et al. (2017), the reference electrode was stimulated for 400 ms, followed by stimulation of the test electrode for another 400 ms. In other studies, stimuli with a maximum duration of 800 ms (Brown et al. 2015; Scheperle & Abbas 2015b) to 1 s (Liang et al. 2018; Han & Dimitrijevic 2020) were used. Our choice of the relatively long duration of the reference tone was based on data we obtained in NH subjects, in which ACC amplitudes following a 3-s pure tone were substantially larger (by a factor ~1.5) than those following a 1-s pure tone (Vonck et al. 2019).

### ACC and Frequency Discrimination

Studies investigating the relationship between ACC measures and frequency discrimination in CI users are scarce. Our findings indicated that frequency discrimination is strongly correlated to the ACC latency, which is supported by Liang et al. (2018) and McGuire et al. (2021). They used pure tone stimuli with a frequency change within the stimulus, just like the ACC stimuli, to obtain frequency change detection thresholds (FCDTs), whereas we measured frequency discrimination thresholds (FDTs) with frequency differences between separate pure tone pips. They found that the N1 latency of the ACC was moderately correlated to the FCDT.

The correlation between ACC and FDT was not as strong for amplitude as for latency, in the sense that amplitude was only correlated to the FDT for the middle but not for the apical electrode. This seems to agree with Liang et al. (2018) who mentioned a statistically non-significant trend that CI users with smaller ACC amplitudes had larger FCDTs (i.e., worse performance). The same group reported in a larger more recent study that ACC amplitude did not correlate with FCDT (McGuire et al. 2021).

### ACC and Stimuli

As expected, stimuli with larger frequency changes resulted in shorter N1 latencies, and larger N1-P2 amplitudes for both the middle and apical electrode (Fig. 3). This has been previously described by our research group (Vonck et al. 2019) and other investigators (Harris et al. 2008; He et al. 2012; Liang et al. 2018; Martin & Boothroyd 2000; Pratt et al. 2009). A neural population responding to the reference tone and a population responding to the target tone will greatly overlap in case of a stimulus with a small frequency change. Thus, only a few neurons that did not respond to the reference tone will respond to the target tone, while the neurons that responded to the reference tone have adapted and will not or only weakly respond to the frequency change. This results in a small overall response. With increasing change in frequency, more neurons respond to the target tone as the overlap in frequency sensitivity decreases. Also the number of neurons specifically responding to the frequency modulation sweep (e.g., Nelken & Versnel 2000) will increase with increasing change magnitude (Vonck et al. 2019). This results in a larger overall response.

The detection of the frequency change in pure-tone stimuli likely relies on cochlear place cues as the result of activating different electrodes (Pretorius & Hanekom 2008; Zhang et al. 2019). We did not conduct loudness balancing within the frequency change stimuli. Thus, since level changes of 2 or 3 dB may evoke an ACC (Martin and Boothroyd 2000; Harris et al. 2007), we cannot exclude that some subjects perceived loudness changes when exposed to a frequency change stimulus. Differences in CI maps may explain differences in ACCs. Therefore, we compared electrodograms (Supplemental Digital Content 1, http://links.lww.com/EANDH/B21) between a subject with large ACCs (BD-03) and a subject with small ACCs (BD-06). Since these electrodograms were quite similar, we assume that at least in these cases individual CI electrode settings were of minor influence on the ACCs.

We conclude that in our study population most successful ACC recordings could be evoked by stimuli with the largest frequency change, which consisted of an increase from the center frequency of the middle or apical electrode towards the center frequency of the third adjacent electrode (i.e., a frequency increase of about 55% or 75%, respectively). In line with literature (Han & Dimitrijevic 2020; Liang et al. 2018) and considering the correlation coefficients (Tables 5 and 6), the ACC latency in response to this stimulus seems to be best correlated to the several speech perception measures in quiet and noise and frequency discrimination, in particular with the apical electrode condition.

### Frequency Discrimination

As expected based on previous studies, speech perception in noise was strongly correlated to frequency discrimination (Fig. 2; He et al. 2012; Turgeon et al. 2015; Zhang et al. 2019). Remarkably, some CI users with high CVC-in-noise scores (>60%) had FDTs comparable to those of NH ears (<2%). Furthermore, many CI users were able to discriminate pure tones within the frequency band of one electrode, which agrees with results reported by Pretorius & Hanekom (2008). They determined FDTs in five postlingually deafened CI users with at least six months of CI experience, using an adaptive two-alternative forced-choice procedure. Discrimination thresholds were within one frequency band in two subjects, while the other three showed FDTs of more than one bandwidth (Pretorius & Hanekom 2008). Supplemental Digital Content 1, http://links.lww.com/EANDH/B21 shows similar electrodograms for a subject who was able to discriminate pure tones within the frequency band of one electrode (BD-03) and a subject who was not (BD-06). Analogous to the ACCs as discussed above in relation to the electrodograms, this implies that an impaired auditory processing underlies the poorer postoperative performance of some CI patients, rather than electrode map settings.

Turgeon et al. (2015) measured the FDT in 20 CI users and 16 NH controls with an adaptive two-alternative forced-choice procedure with pure reference tones of 500 or 4000 Hz. Based on speech perception in quiet, CI users were categorized in ten good performers (word recognition score > 65%) and ten poor performers (word recognition score < 65%). In line with our findings, their FDTs were moderately correlated with speech perception in quiet and the good performers tended to have comparable FDTs to the NH subjects (Turgeon et al. 2015). Corresponding to these outcomes, Zhang et al. (2019) found that FCDTs of 20 CI users were strongly correlated to speech perception (i.e., word recognition in quiet, sentence recognition in quiet and noise, and digits-in-noise).

Taken together, psychophysical frequency discrimination tests have a moderate to strong correlation with speech perception and could be used as a non-linguistic estimate of auditory performance.

### Single-Sided Deafness and Cochlear Implantation

In NH subjects, unilateral stimulation results in asymmetrical activation of the central auditory system with more excitatory activity at the contralateral hemisphere than at the ipsilateral hemisphere, which is called (normal) hemispheric asymmetry (Hine & Debener 2007; Maslin et al. 2013b). Maslin et al. (2013b) compared multi-channel CAEPs of 18 subjects with acquired unilateral deafness, after translabyrinthine surgery for the removal of an acoustic neuroma, to 18 NH controls. They reported that in the SSD subjects stimulation of their only hearing ear led to increased N1 amplitudes in the ipsilateral hemisphere, while the activity in the contralateral hemisphere remained largely unchanged. So, monaural stimulation in individuals with late onset SSD results in a greater overall response of the central auditory cortex and reduced hemispheric asymmetry, which has also been described by Ponton et al. (2001). This experience related plasticity seems to occur within 1 month post-onset of SSD, and continues for at least 6 months (Maslin et al. 2013a). These findings are consistent with adult animal models with experimentally induced unilateral deafness, showing greatly enhanced excitatory activity in the ipsilateral inferior colliculus and primary auditory cortex evoked by stimulation of the hearing ear, which was found to be a consequence of reduced inhibition (Moore et al. 1997; Mossop et al. 2000; Popelár̆ et al. 1994).

Cochlear implantation for SSD has an effect on these changes in the auditory cortex. Legris et al. (2018, 2019) investigated the CAEP (evoked by speech-in-noise stimuli, binaurally presented in the free field) in eight NH controls and in nine SSD subjects before cochlear implantation, and 6 and 12 months after implantation. The CAEP amplitude at the mastoid contralateral to the deaf ear increased within SSD subjects after cochlear implantation. CAEP latencies, however, remained significantly longer for some components at temporal and mastoid sites than for NH controls, even after cochlear implantation. The observed increased bilateral cortical activation after cochlear implantation, together with an improvement in functional speech in noise testing, may indicate the restoration of binaural cortical function (Legris et al. 2018).

Human studies are complemented by animal models of CI use that reveal mechanisms of adaptation and plasticity. In bilaterally congenitally deaf cats, it was observed that unilateral hearing experience with a CI leads to cortical activity that is boosted due to the lack of contralateral inhibition normally involved in bilateral hearing (Gordon et al. 2013; Kral et al. 2013a; Syka 2002). Tillein et al. (2016) investigated cortical responses to CI stimulation in unilaterally and bilaterally congenitally deaf cats, and found that, compared to bilateral deafness, unilateral deafness enhanced aural dominance of the NH ear.

Based on these studies, one could expect the ACC amplitudes contralateral to the CI ear to be smaller in SSD subjects than in unilaterally implanted BD subjects, as a consequence of the inhibitory input from the NH ear. On the other hand, one could expect the amplitudes contralateral to the CI ear to be larger in SSD subjects than in unilaterally implanted BD subjects, as a result of sustained overall cortical responsiveness in SSD subjects caused by auditory input of their NH ear, as shown by Tillein et al. (2016). Our adult SSD subjects, all six with late onsets of their SSD (age > 40 years, see Table 1), have presumably established bilateral auditory pathways with normal cortical organization until the onset of SSD (Kral et al. 2013b). However, no significant differences in ACC latencies or amplitudes were found between the CI ears of the unilaterally and bilaterally deaf subjects. Considering the small sample size of six SSD subjects, which limits statistical power, one should note that a trend toward a difference was not observed.

To our knowledge, this study is the first to report ACC measures in SSD subjects. We found longer ACC latencies and smaller amplitudes for the CI ear compared to latencies and amplitudes for the NH ear. However, careful interpretation is essential, because this could also be the result of the different methods we used for presenting stimuli to the CI ear (i.e., electrically, via a direct audio input cable) and NH ear (i.e., acoustically, via a headphone). Wedekind et al. (2020) presented speech tokens in the free field to the CI and NH ear of 29 SSD patients and found quite similar CAEPs to these onset stimuli. This is in contrast to the electrophysiological differences between the CI ear and NH ear of SSD subjects found in our study and reported by Tillein et al. (2016). Wedekind et al. (2020) investigated the N1 latency of the CAEP ipsilateral and contralateral to the CI and NH ear, and found no significant difference in latencies between those recordings, indicating that sound-evoked activity in the auditory cortex occurred simultaneously (Wedekind et al. 2020). Although their study population was quite comparable to ours in terms of duration of deafness and implant experience, the CI brands and, most importantly, the used stimuli differed (onset of speech token versus frequency change in pure tone).

Our sample size of SSD subjects was relatively small. Therefore, unsurprisingly, we found no correlation between the differences in ACC measures of the individual CI ear and the NH ear, and frequency discrimination or speech perception in noise (mono-aurally tested), after at least 1.4 year of CI experience. Nevertheless, we observed that subjects SSD-05 and SSD-06 showed a large difference in latency between their CI ear and NH ear with the apical electrode condition (Fig. 8) and that they both had the worst speech perception in noise in the SSD group (Table 2). Regarding the ACC amplitude, subjects SSD-02 and SSD-05 showed a large difference in amplitude between their CI ear and NH ear with the middle electrode condition (Fig. 8). They both had the longest duration of deafness in the SSD group (8.3 and 4.3 years, respectively), which possibly resulted in lasting aural dominance of the NH ear, thus explaining the large amplitude differences.

### Clinical Implications and Future Direction

Psychophysical frequency discrimination tests correlate well with speech perception in noise in both traditional bilaterally deaf CI users as well as SSD patients. Therefore, the ACC as an objective measurement indicative for hearing performance can be valuable in the management of CI users. In particular, the ACC provides information on hearing performance in CI users who cannot reliably perform behavioral tasks, for example. poor-performing CI users, young children, in case of cognitive impairment, or a language barrier. In these patients, the assessment of hearing performance by the ACC can aid in the auditory rehabilitation after implantation. Based on our findings, and to make the ACC clinically applicable and less time-consuming, limiting to a maximum of 45 minutes, we recommend using a pure tone with a large frequency change (e.g., +3 electrode step, corresponding to a frequency increase of about 55% or 75% compared with the reference frequency of the middle or apical electrode).

Recently, the ACC was measured in NH musicians and non-musicians to investigate the effects of long-term musical training (Brown et al. 2017). The ACC amplitudes were larger in the group of musicians, which suggested that the ACC might be useful as an outcome measure in exploring the efficacy of interventions as musically based, auditory training programs, which may also be applied to CI users as indicated in a recent pilot study (Firestone et al. 2020). Furthermore, some investigators have already shown that it is possible to record the ACC with speech stimuli in NH infants and young children (Chen & Small 2015; McCarthy et al. 2019; Small & Werker 2012; Uhler et al. 2018), and also in children with hearing loss (Martinez et al. 2013).

Previous research in animals, children, and adults revealed that cortical responses undergo significant morphological changes during the first months after CI activation (e.g., Gordon et al. 2015; Kral et al. 2013b; Legris et al. 2018; Maslin et al. 2013a; Mathew et al. 2018). Thus, evaluating the ACC before and during the first months after implantation is relevant to perceive insights in cortical reorganization and central auditory processing. Such insights aid in prognosis and planning of auditory training programs for the CI user.

## CONCLUSION

In this study we examined the ACC as an objective measure for hearing performance in both traditional bilaterally deaf and in single-sided deaf CI users. The results suggest that the ACC latency and amplitude evoked by tone frequency changes correlate well to the postoperative performance and that it can be of added value in assessing frequency discrimination and speech perception capabilities. We found no difference in perceptual or electrophysiological outcomes of CI ears between bilaterally and single-sided deaf subjects.

## ACKNOWLEDGMENTS

B.M.D.V., H.V., and M.J.W.L. designed the study; J.A.A.v.H. and B.M.D.V. acquired the data; J.A.A.v.H analyzed the data; J.A.A.v.H wrote a significant portion of the article and made figures; B.M.D.V., R.J.S., H.V., and M.J.W.L. provided critical revision of the manuscript. All authors provided final approval of the version to be published.

## Supplementary Material


